# Modifications of Furan-Based Polyesters with the Use of Rigid Diols

**DOI:** 10.3390/polym16142064

**Published:** 2024-07-19

**Authors:** Konrad Walkowiak, Sandra Paszkiewicz

**Affiliations:** Faculty of Mechanical Engineering and Mechatronics, West Pomeranian University of Technology, 70-310 Szczecin, Poland; sandra.paszkiewicz@zut.edu.pl

**Keywords:** copolyesters, furan-based polyesters, two-step melt polycondensation, rigid diols, isosorbide, CBDO, CHDM

## Abstract

The replacement of polymers derived from petrochemical resources has been a prominent area of focus in recent decades. Polymers used in engineering materials must exhibit mechanical strength and stiffness while maintaining performance through a broad temperature range. Most of the polyesters used as engineering materials are based on terephthalic acid (TPA) and its derivatives, which provide necessary rigidity to molecular chains due to an aromatic ring. Bio-based alternatives for TPA-based polyesters that are gaining popularity are the polyesters derived from 2,5-furandicarboxylic acid (FDCA). To broaden applicational possibilities, one effective way to achieve specific properties in targeted applications is to adjust the composition and structure of polymers using advanced polymer chemistry techniques. The incorporation of rigid diols such as isosorbide, 1,4-cyclohexanedimethanol (CHDM), and 2,2,4,4-tetramethyl-1,3-cyclobutanediol (CBDO) should result in a greater stiffness of the molecular chains. This review extensively explores the effect of incorporating rigid diols on material properties through a review of research articles as well as patents. Moreover, this review mainly focuses on the polyesters and copolyesters synthesized via two-step melt polycondensation and its alterations due to the industrial importance of this method. Innovative synthesis strategies and the resulting material properties are presented.

## 1. Introduction

**Figure 1:**
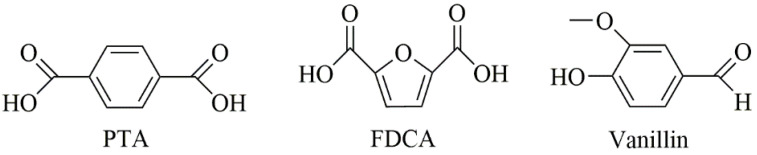
Chemical structure of PTA, FDCA, and vanillin.

If the current growth trends continue, the annual global production of plastics is projected to increase to 1124 million metric tons by 2050 [1,2]. Currently, a majority of these polymeric materials are fossil-based monomers, which are heavily reliant on the consumption of petrochemical resources. Faced with the challenges of dwindling fossil fuel reserves and the environmental persistence of fossil-based polymers, there is a growing momentum to innovate and embrace sustainable resources. Biomass stands out as an increasingly attractive alternative to oil and coal, primarily because it is one of the few widely accessible sources of carbon. Biomass includes various sources such as carbohydrate feedstocks, lignin, vegetable oils, and more [3]. Nowadays, the production capacity of bio-sourced polymers has reached 12 million metric tons annually [1]. Nevertheless, most of the currently produced bio-based polymers, like polylactic acid, polybutylene succinate, or polyglumatic acid, have an aliphatic structure, while petroleum-based engineering plastics have mostly an aromatic structure. This disparity underscores the importance of developing bio-based materials with aromatic structures, a crucial step in replacing traditional petroleum-based polymers. The most discussed bio-based aromatic monomers are (purified) terephthalic acid (PTA), 2,5-furandicarboxylic acid (FDCA), and vanillin, which are shown in Figure 1.

Over the past few years, FDCA preparation and purification technologies have constantly evolved [4,5,6,7]. The FDCA has various synthesis methods, however, the most promising route is synthesis from 5-hydroxymethylfurfural (HMF). In this route, the cellulose or cellulose-derived carbohydrates are first dehydrated to HMF. Subsequently, the hydroxymethyl group of HMF is then oxidized to an aldehyde group, which is further oxidized to carboxyl to produce FDCA. FDCA was selected as one of the twelve most promising bio-based platform compounds by the U.S. Department of Energy and stands out as the sole bio-based aromatic chemical on this list. FDCA has a similar structure to that of PTA (Figure 1). Its structural resemblance to PTA, as illustrated in Figure 1, positions FDCA as an ideal bio-based alternative for PTA, which is currently predominant in polymer and resin production. Given the diversity and accessibility of bio-based linear diol units with varying carbon atoms, researchers have developed a range of FDCA-based polyesters. The most representative and well-analyzed FDCA-based polyester is poly(ethylene 2,5-furandicarboxylate) (PEF), which is an alternative to commercially available poly(ethylene terephthalate) (PET). PEF can be synthesized using conventional polymerization routes, such as direct esterification [8], transesterification [9], polytransesterification [10], and ROP [11]. The reports considering PEF show that it has more attractive thermal and mechanical properties than PET [12]. PEF also has superior barrier properties, its oxygen permeability is 10 times lower, and its CO_2_ permeability is 19 times lower than PET [13,14]. Higher thermal, mechanical, and barrier properties of PEF, when compared to PET, result from the rigid structure of the furan ring and suppressed furan ring-flipping [15]. Moreover, the crystallization rate of PEF is slower when compared to PET due to the lower molecular mobility of PEF caused by the furan ring. A particular effort has been placed on precisely tailoring the composition and structure of polymers through innovative polymer chemistry methods. In order to increase the rigidity of the molecular chain and obtain excellent thermomechanical properties, the use of rigid diols is becoming more common.

**Figure 2:**
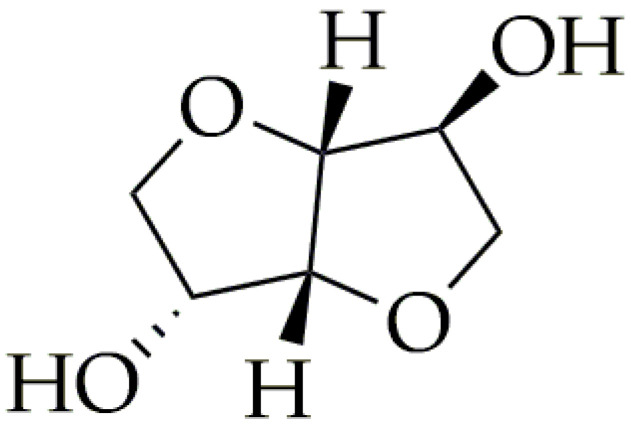
The chemical structure of isosorbide.

Currently, a majority of fossil-based monomers, such as terephthalic acid [16,17,18,19,20,21,22], aliphatic acids [23,24,25,26,27,28,29,30,31], and diols [32,33,34,35,36], are capable of being synthesized from bio-based feedstocks. However, the key to achieving improved material properties that were not available before lies in new monomers derived from bio-based sources. Isosorbide is an important bio-based monomer for high-performance materials. The synthesis route of isosorbide starts from the hydrogenation of glucose to sorbitol. Subsequently, it is exposed to acidic conditions, which results in a selective double-dehydration process. This critical step is pivotal, as it effectively produces isosorbide [37]. Polymers based on isosorbide possess excellent properties such as a strong resistance to UV radiation, heat, chemical degradation, and tensile properties [37]. Isosorbide can be used in the production of polyesters, polycarbonates, and polyurethanes. However, despite the excellent benefits of adding isosorbide to polymers, it can prove to be difficult. Isosorbide has low reactivity, due to the fact that it is a secondary diol (Figure 2). Therefore, polymers based on isosorbide have a low isosorbide content (>50 mol% isosorbide as a percentage of the total diol) [38,39,40]. The addition of a higher content of isosorbide results in low molecular weights and longer times of synthesis. Despite this, isosorbide found its way to commercial applications. In 2014, a significant portion, 35%, of the globally produced isosorbide was used to produce poly(ethylene-co-isosorbide terephthalate) (PEIT) [41]. The properties of PEIT depend on the isosorbide content. When isosorbide is added in small amounts (below 20 mol.%), it allows for obtaining semicrystalline copolymers with higher thermal stability than PET. Thus, PEIT can find applications such as heat-resistant fibers [42]. On the other hand, incorporating isosorbide in larger amounts (over 20 mol.%) hinders the crystallization process, which results in an amorphous structure of PEIT. The value of its glass transition temperature (T_g_) is more than 100 °C (varying with the isosorbide content). With more than 20 mol.% isosorbide, PEIT’s properties can compete with other amorphous polymers such as polycarbonates (PC) or poly(methyl methacrylate) (PMMA). This perspective of the utilization of isosorbide in the industry is also reflected in the market. In 2018, the global market for isosorbide stood at a notable USD 190 million. Moreover, it is projected to reach USD 350 million by 2023, indicating a significant growth trajectory in this sector [43].

**Figure 3:**
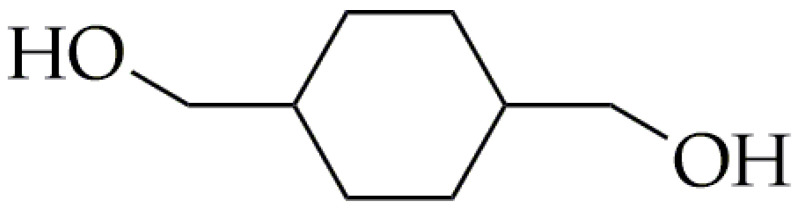
The chemical structure of CHDM.

Another important rigid diol is 1,4-cyclohexanedimethanol (CHDM). CHDM is an alicyclic diol (Figure 3), which was developed at Tennessee Eastman Co. (Kingsport, TN, USA), a division of Eastman Kodak Co. (Rochester, NY, USA) [44]. Moreover, the CHDM molecule effectively serves as a linking agent in the production of resins and fibers, such as polycarbonates and polyurethanes, which exhibits excellent thermal resistance and enhanced physical strength [45,46,47]. CHDM is commercially synthesized in two steps. During the first step, catalytic hydrogenation of dimethyl terephthalate (DMT) is performed to obtain dimethyl 1,4-cyclohexanedicarboxylate (DMCD) [44,48]. In the second step, DMCD undergoes reduction using copper chromite, resulting in commercial CHDM that is typically a mix of isomers with a predominant 70/30 trans-to-cis ratio. The ratio of isomers in CHDM is crucial in determining the final properties of CHDM-containing copolyesters, as it affects the efficiency of chain packing [48]. The content of the CHDM in the copolymers has a significant effect on the crystalline structure; the rigid structure of this diol hinders crystallization. The incorporation of the low-content CHDM (less than 5 mol%) into poly(ethylene terephthalate) (PET) results in the production of stretch blow-molded containers for carbonated soft drinks. This is because CHDM alters the material’s stretching behavior, consequently widening the processing window. The amorphous copolyesters obtained by the addition of a sufficient amount of CHDM into PET were first evidenced by the Kibbler patent in 1959 [49]. However, Eastman did not commercialize CHDM-modified PET (PET-G) until 1977 [44]. Nowadays, PET-G has various applications, including being used as a filament for 3D printing, extrusion blow molding, and sheet extrusion. CHDM has also found industrial use, in the production of Tritan^®^ by the Eastman Chemical Company since 2007. Other monomers used in the synthesis of Tritan^®^ include dimethyl terephthalate (DMT) and 2,2,4,4-tetramethyl-1,3-cyclobutanediol (CBDO).

**Figure 4:**
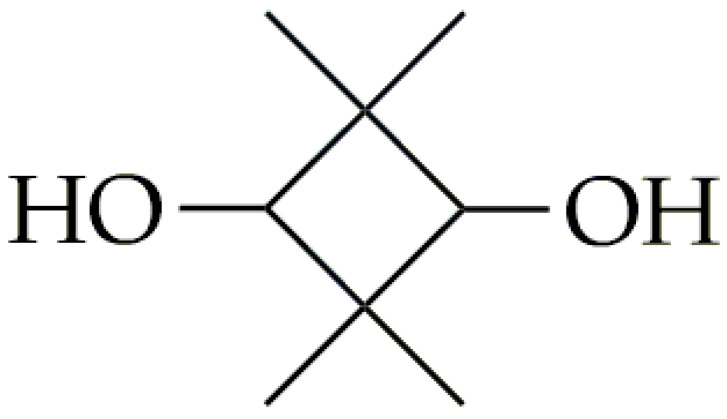
The chemical structure of CBDO.

CBDO, another rigid diol, has its chemical structure presented in Figure 4. This monomer has a cyclic aliphatic structure, which is used for the synthesis of high-performance functionalized polyester materials [50,51]. CBDO is synthesized by the hydrogenation of 2,2,4,4-tetramethyl-1,3-cyclobutanedione (CBDK) in a dihydrogen atmosphere [51]. Efficient, selective, and stable hydrogenation catalysts are crucial for large-scale CBDO production. Various supporting catalysts, including those based on Pd [52], Ru [53,54], Pt [55], Rh [56], and the more affordable Cu and Co [57], have been investigated for CBDO hydrogenation reactions. Among these, Ru-based catalysts have proven to be promising for practical applications.

Numerous reviews on polyesters derived from FDCA and its derivatives have been published in recent years [40,58,59,60,61]. Additionally, several papers have focused on the properties and recycling of furan-based polyesters, such as [62,63,64]. However, there is a lack of reviews addressing the synthesis of copolymers through two-step melt polycondensation using rigid diols and FDCA derivatives. Melt polycondensation is extensively employed in the production of traditional polyesters such as PET and polybutylene terephthalate (PBT). The detailed information about this synthesis method of furan-based materials underscores future industrial applications of these materials.

## 2. Polyesters and Copolyesters Based on FDCA

### 2.1. Polyesters Based on FDCA and Its Derivatives

**Figure 5:**
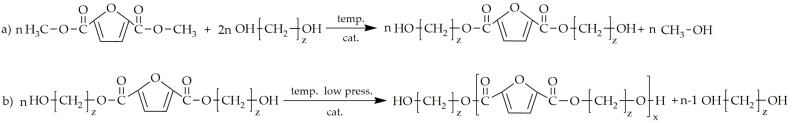
The synthesis of furan-based polyesters with the use of DMFD and diols: (**a**) transesterification, (**b**) polycondensation.

The first synthesis of a furan-based polyester was recorded in 1946 by the Celanese Corporation of America, which submitted a patent for the synthesis of PEF using FDCA [65]. The rise in the intensity of research work on obtaining polyesters based on FDCA and its derivatives did not occur until the 21st century. There has been a surge in interest, evidenced by significant investments in two major European initiatives focused on researching and potentially commercializing FDCA-based polyesters. These initiatives include PEFerence (project number 744409), which is supported by Horizon 2020, the European Union’s flagship research and innovation program, and the COST Action FUR4Sustain (CA18220), which is part of the European Cooperation in Science and Technology framework. This sudden growth of interest is due to the fact that the methods for preparing and purifying FDCA have seen significant advancements. Among the various methods available for synthesizing FDCA, the most efficient and promising is conversion from 5-hydroxymethylfurfural (HMF) [66]. This process begins with the dehydration of cellulose or cellulose-based carbohydrates to form HMF. Following this, the hydroxymethyl group in HMF undergoes oxidation to transform into an aldehyde group. This aldehyde group is then further oxidized into a carboxyl group, culminating in the production of FDCA. A majority of furan-based polyesters are synthesized via two-step melt polycondensation, which is presented in Figure 5. The first step of this process is esterification or transesterification, which is followed by polycondensation. Polyesters are more frequently synthesized using dimethyl-2,5-furandicarboxylate (DMFD) instead of FDCA, primarily due to the easier purifying of DMFD through recrystallization from methanol. This process ensures a higher purity of this monomer. Additionally, a synthesis using DMFD can be conducted under milder conditions compared to a synthesis that utilizes FDCA [67]. 

Academia has taken up the challenge of synthesizing furan-based polyesters utilizing linear diols with a different number of carbon atoms (C3–20) [40]. The most popular furan-based polyesters (except the abovementioned PEF) are poly(propylene 2,5-furandicarboxylate) (PPF) and poly(butylene 2,5-furandicarboxylate) (PBF). The reason for this is that, as with PEF, PPF and PBF are homologs for the commercially available poly(propylene terephthalate) (PPT) and poly(butylene terephthalate) (PBT). According to [8,12], PPF has a higher value of T_g_ compared to PPT and very similar values of the beginning of the thermal decomposition temperature and temperature at the maximum degradation rate (T_d,max_). Moreover, PTF has higher barrier properties when compared to PEF [68]. PPF exhibits excellent mechanical properties, with a Young’s modulus (E) of around 1.6–2.7 GPa, a tensile stress at break (σ_b_) 67–82 MPa, and elongation at a break (ε_b_) of around 3% [1,8,69]. PPF can be used for packing but also in other fields like sensors or electronics [70]. The functional properties of PBF were found to be comparable to those of PBT [71,72,73]. The T_g_ value of PBF is around 31–46 °C and exhibits a melting temperature of about 168–172 °C [8,70,73,74]. The E value of PBF is reported to be at 742–1000 MPa, while the σ_b_ is approximately at 5.5–31.8 MPa, and elongation occurs at a break of around 2.5–1184% [7,71]. Moreover, PBF has two crystalline structures (α phase and β phase) that are similar to the crystal structure of PBT [70]. The length of the linear diol units used for the synthesis of FDCA-based polyesters influences thermal and mechanical properties. Along with the increasing chain length, one can observe the decrease in the T_g_, σ_b_, and E values. Furthermore, the value of ε_b_ increases [8,67,75,76,77]. The degree of crystallinity increases with a greater distance between the furan rings, which allows for higher mobility and results in a higher regularity of the molecular chain [78]. The melting temperature (T_m_) is also influenced by the length and the structure of the diol units; with an increasing length, the value of T_m_ decreases.

### 2.2. Polyesters and Copolyesters Based on FDCA and Isosorbide

**Figure 6:**
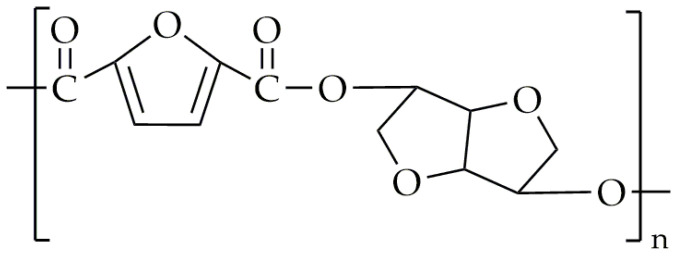
The chemical structure of PIF.

The synthesis of FDCA polyesters, which contain isosorbide units, has been proven to be difficult [38,39]. This is due to the fact that isosorbide is a secondary diol, which results in low reactivity. The first attempt to solve this problem for FDCA-based copolyesters which contain isosorbide was made in 1993 by Storbeck et al. [79]. Instead of using FDCA for the synthesis, 2,5-furandicarbonyl dichloride was used, and they did not limit themselves only for the isosorbide but for all three isomers of 1,4:3,6-dianhydrohexitol. The solution polycondensation took place in 1,1,2,2-tetrachloroethane in the presence of pyridine. The T_g_ values of the obtained polyesters were within the range 173–194 °C, while the intrinsic viscosities were in the range of 0.11 dL/g and 0.38 dL/g. The intrinsic viscosity of the PET used for the production of bottles is between 0.7 dL/g and 0.78 dL/g [80], which is significantly higher than obtained results for the mentioned polyesters. However, the main scope of this review is the synthesis of polyesters and copolyesters via melt polycondensation. Several attempts were made to synthesize poly(isosorbide 2,5-furandicarboxylate) (PIF) [81,82], aiming to achieve high T_g_ and intrinsic viscosity values. The chemical structure of PIF is presented in Figure 6. Wang et al. [81] performed melt polycondensation using DMFD and isosorbide as the monomer and the as catalyst, and they chose COOMe/OH 1:1.6. However, the intrinsic viscosity of the synthesized material was only about 0.27 dL/g, while its T_g_ value was 162 °C. PepsiCo, Inc. (New York, NY, USA) [82], in 2013, patented the synthesis of PIF using FDCA and isosorbide as the monomer and Sb_2_O_3_ as the catalyst. The esterification was conducted in two steps: (1) The mixture was heated to 220–230 °C and maintained at this temperature for 10 h. (2) The temperature was then increased to 260 °C and maintained for an additional 10 h. After that, a vacuum was applied to remove any water. The PIF synthesized by PepsiCo, Inc. had a T_g_ value of around 137 °C. Unfortunately, the molecular weight or intrinsic viscosity value was not provided. Terzopoulou et al. [83] modified the procedure of melt polycondensation for the synthesis of PIF. The transesterification was performed with a temperature range of 150 °C to 170 °C for 4 h under an argon atmosphere. After that, another 1.05 equivalents of DMFD were added. The reaction between the newly added DMFD and the mixture in the reactor lasted 5 h at 150–170 °C in an argon atmosphere. The polycondensation was carried out for 3 h within the temperature range of 210 °C to 230 °C under low-pressure conditions. The main objective of this modification was to obtain a high molecular weight of the PIF. The intrinsic value of the synthesized PIF was 0.39 dL/g, and the T_g_ value was about 157 °C.

**Figure 7:**
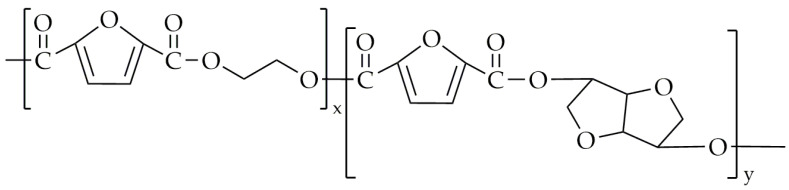
The chemical structure of PEIF.

Moving to copolyesters based on FDCA and its derivatives, the best studied FDCA-based polyester is PEF, and there are already published papers and patents attempting a synthesis of poly(ethylene-co-isosorbide-2,5-furandicarboxylate) (PEIF) with a high value of molecular mass, intrinsic viscosity, and T_g_ with the use of the traditional route of two-step melt polycondensation. The chemical structure is presented in Figure 7. PEIF was patented in US20130171397A1 by PepsiCo, Inc. [82], with an isosorbide content of 0.7%, 2%, 5%, and 10%. The highest observed value of T_g_ was 78 °C for PEIF 99.3%EG/0.7IS, while the lowest T_g_ value was 64 °C for PEIF with 95%EG/5%IS. Compared to the synthesized PEF, which had a T_g_ of 79 °C, it can be concluded that the addition of isosorbide in this case did not increase the T_g_. The degree of crystallinity (X_c_) for PEIF was between 20–25%.

The other patent, WO2015142181A1, by Roquette Frères was published in 2015 [84]. This synthesis was performed in a 200 mL glass reactor and a 2 L stainless-steel reactor. Furthermore, DMFD was used instead of FDCA as the monomer, and in most cases, titanium tetrabutoxide was used as the catalyst. In a glass reactor, the transesterification was conducted at 160–190 °C, while polycondensation was carried out at up to 240 °C for 210 min at a pressure reduced to 5 mbar. In a 2 L stainless-steel reactor at the beginning of the reaction, vacuum–nitrogen cycles were performed at 60–80 °C at a constant string of 20 rpm. The transesterification was carried out at 130–190 °C, under nitrogen pressure, and stirred at the rate of 150 rpm. The polycondensation process was conducted at temperatures ranging from 235 °C to 270 °C and lasted between 100 min and 255 min under low-pressure conditions. The results are presented in Table 1**.**

Moreover, a modified route of this synthesis was presented in WO2015142181A1. Firstly, the authors carried out the transesterification between isosorbide/ethylene glycol and DMFD at a molar ratio of 1:2.1 or 2.5:1, respectively. This procedure involved heating the mixture to 190 °C for 2 h when isosorbide was added or for 3 h when ethylene glycol was added. After that, isosorbide/ethylene glycol was added, and the mixture was kept for an additional 2 h. The polycondensation was carried out at 240 °C for 4 h under low-pressure conditions. The results are presented in Table 2.

**Figure 8:**
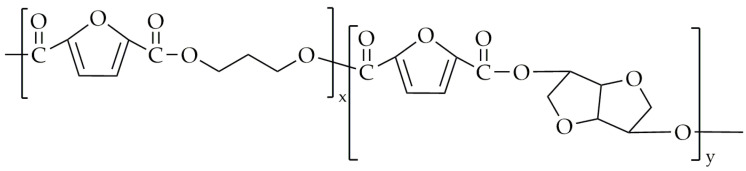
The chemical structure of PPIF.

The authors of this work attempted the synthesis of poly(propylene-co-isosorbide-2,5-furandicarboxylate) (PPIF) via two-step melt polycondensation. As monomers, we used DMFD (Henan Coreychem Co., Ltd., Zhengzhou, China); dimethyl isosorbide (Sigma-Aldrich, Saint Louis, MO, USA); and 1,3-propanediol (bio-PDO, DuPont Tate & Lyle BioProducts, London, OH, USA). The transesterification was conducted at a temperature range of 165 °C to 195 °C for up to 4 h. The polycondensation was performed at up to 235 °C under low-pressure conditions. Dimethyl isosorbide was added in molar fractions at 15 mol.% and 25 mol.%. The chemical structure of PPIF is shown in Figure 8.

**Figure 9:**
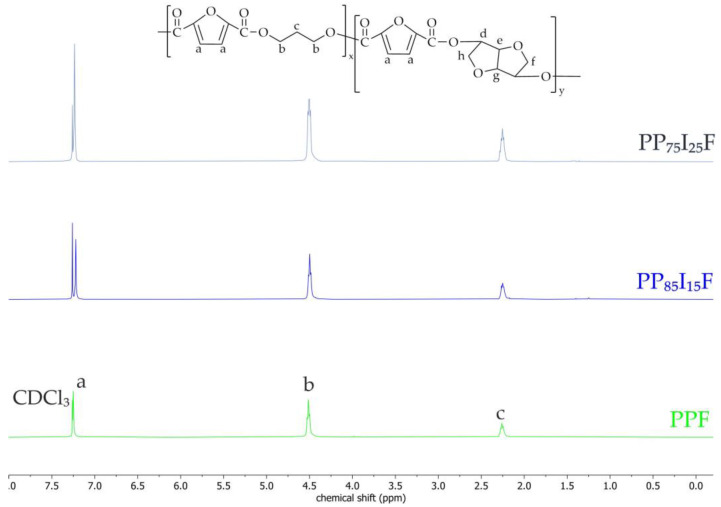
^1^H NMR spectra of PPF and PPIF.

The proton nuclear magnetic resonance (^1^H NMR) was carried out on a spectrometer operating at a frequency of 400 MHz (Bruker, Karlsruhe, Germany). Before the experiment, all samples were subjected to continuous methanol extraction for 24 h. All materials were dissolved in chloroform-d CDCl_3_ at 10 mg/mL. The spectrometer operated at 400 MHz, and tetramethylsilane (TMS) was used as an internal chemical shift reference. The obtained spectra are presented in Figure 9. Unfortunately, an analysis of NMR spectroscopy showed that the isosorbide units were not incorporated into the polymer chain.

**Figure 10:**
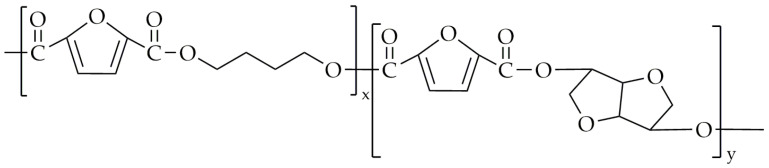
The chemical structure of PBIF.

Wang et al. [81] attempted to synthesize poly(1,4-butylene-co-isosorbide-2,5-furandicarboxylate) PBIF using traditional two-step melt polycondensation. The chemical structure is presented in Figure 10. The transesterification was performed at 200 °C for 2 h, followed by an additional hour at 210 °C. The polycondensation lasted 6 h at 250 °C under low-pressure conditions. The synthesized copolyesters contained 20 mol% to 80 mol% isosorbide units. The properties of copolyester can be found in Table 3.

With an increase in the isosorbide content, a decrease in the value of M_n_ and intrinsic viscosity was observed. The incorporation of the isosorbide resulted in an amorphous structure. Furthermore, a significant increase in the T_g_ value is observed; the PBIF with 80 mol.% of isosorbide content had an almost 3-times higher T_g_ value when compared to the PBF. A higher content of isosorbide units resulted in an increased value of σ_b_, with PBIF containing 70% isosorbide units reaching the highest value of 140 MPa, which is 87 MPa higher than that of PBF. However, the ε_b_ value decreased with a higher content of isosorbide. The PBIF with 80% isosorbide units exhibited the lowest ε_b_ value at approximately 15%, in contrast to PBF, which exhibited an ε_b_ value of around 685%. The E value decreased with up to 50% of isosorbide content, but beyond that, the E value of copolyesters began to exceed that of PBF.

**Figure 11:**
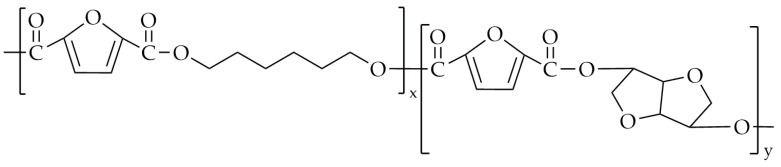
The chemical structure of PHIF.

The chemical structure of poly(1,6-hexamethylene-co-isosorbide-2,5-furandicarboxylate) (PHIF) is shown in Figure 11.

**Figure 12:**
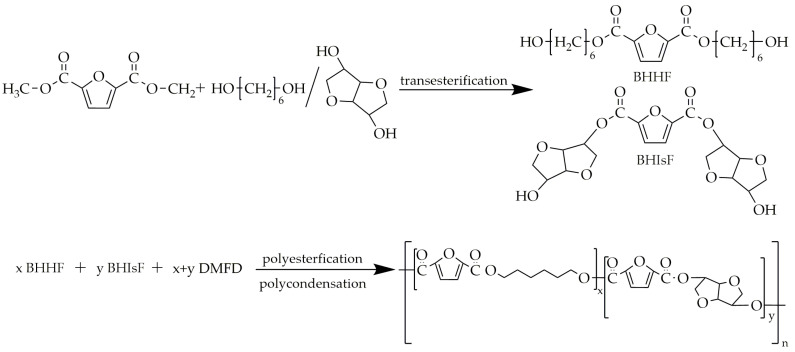
The scheme of the synthesis of PHIF.

Kasmi et al. [85] synthesized PHIF using DMFD, 1,6-hexanediol (HDO), and isosorbide as monomers and titanium (IV) isopropoxide as the catalyst. The copolyesters were synthesized utilizing three-step melt polycondensation. The first step was transesterification between DMFD and HDO/isosorbide in order to obtain bis(hydroxylhexamethylene)-2,5-furan dicarboxylate (BHHF) and bis(hydroxyisosorbide)-2,5-furan dicarboxylate (BHIsF). this transesterification lasted 4 h over a temperature range of 160 °C to 180 °C. The next step involved the polyesterification of BHHF and BHIsF with an equimolar addition of DMFD, with the ratio of BHIsF varying from 3% to 90%. This polyesterification was carried out in the range of temperatures from 160 °C to 190 °C for about 4.5 h. The last step, polycondensation, was conducted under low-pressure conditions, with a temperature range of 220–250 °C, and lasted 3.5 h. The scheme of this synthesis is presented in Figure 12.

The properties of the selected copolyesters are summarized in Table 4. The intrinsic viscosity values of the obtained copolyesters ranged from 0.23 dL/g to 0.58 dL/g. PHIF 10/90 has the lowest intrinsic viscosity value while PHIF 90/10 has the highest. PHF exhibited an intrinsic viscosity of around 0.40 dL/g. This indicates an improvement in the method, as copolyesters with up to 40% isosorbide units have a similar intrinsic viscosity to neat polyester. The incorporation of the isosorbide unit resulted in a significant increase in the T_g_ value. PHIF 90/10 exhibited the highest T_g_ value at 135 °C, which is approximately 128 °C higher than that of neat PHF. Moreover, the addition of isosorbide resulted in a decrease in the value of X_c_, above 10% of the isosorbide unit content of the copolyesters, which were completely amorphous. The copolyesters exhibited a higher value of decomposition temperatures at a 5% weight loss (T_d,5%_) when compared to the PHF. PHIF 90/10 had a T_d,5%_ value of approximately 373.5 °C compared to neat PHF, which had a T_d,5%_ value of 339.7 °C. This is due to the higher thermal stability of the rigid diols compared to aliphatic diols.

**Figure 13:**
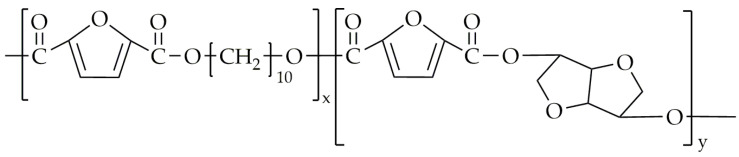
The chemical structure of PDIF.

Another copolyester synthesized by the same group was poly(decamethylene-co-isosorbide-2,5-furandicarboxylate) (PDIF) [86], with its chemical structure shown in Figure 13.

The synthesis of PDIF was nearly identical to that of PHIF, with the exception that 1,10-decanediol was used instead of 1,6-hexanediol. The content of the added isosorbide unit ranges from 5 mol.% to 40 mol.%. The properties of PDIF can be found in Table 5. As with the other copolyesters, the incorporation of isosorbide resulted in a gradual decrease in the values of M_n_ and M_w_. However, the highest decrease was observed for PDIF 70/30 instead of PDIF 60/40. The addition of the 5 mol.% and 10 mol.% of isosorbide units resulted in a decrease in T_g_ when compared to the neat poly(decamethylene 2,5-furandicarboxylate) (PDF). The highest value of T_g_ was exhibited by PDIF 60/40 (20.6 °C). Unlike the most copolyester-containing isosorbide, all PDIF copolyesters have a semicrystalline structure with an X_c_ value ranging from 29.2% to 23.2%. However, in contrast to PHIF copolymers, the incorporation of isosorbide units into PDIF does not significantly increase the value of T_d,5%_, exhibiting an increase of only up to 6.7 °C. The addition of up to 15 mol% isosorbide units resulted in higher E, σ_b_, and ε_b_ values compared to PDF. Beyond a 15 mol% isosorbide content, significant decreases in E and σ_b_ values were observed. This effect is due to the lower values of molecular weight and X_c_. Furthermore, copolyesters with an isosorbide content of up to 20 mol% were subjected to soil burial for 24 weeks. The visible change in weight of the sample was observed at 16 weeks of the experiment. However, the higher the isosorbide content, the lower the weight loss of the sample.

**Figure 14:**
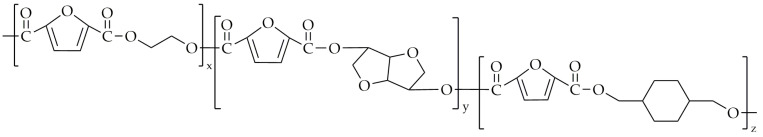
The chemical structure of PEICF.

Kim et al. [87] attempted the synthesis of poly(ethylene-co-isosorbide-co-1,4-cyclohexanedimethylene-2,5-furandicarboxylate) (PEICF), whose chemical structure is presented in Figure 14.

The terpolysters were synthesized with the use of two-step melt polycondensation. Before the esterification, the monomers were melted in a reactor at 110–150 °C under mechanical stirring. This lasted until the mixture was completely melted, after which a catalyst (titanium butoxide) was added. The esterification was carried out at 230 °C, and it lasted for 2 h. The polycondensation was performed at 250 °C, under low-pressure conditions, until the torque reached 30 Nm. The content of the EG in all copolyesters was 50 mol.%, the CHDM unit varied from 19 mol.% to 51 mol.%, and the isosorbide unit was in range of 12 mol.% to 49 mol.%. The properties of terpolysters can be found in Table 6. The M_n_ value did not differ significantly between copolymers. Poly(ethylene-co-1,4-cyclohexanedimethylene-2,5-furandicarboxylate) (PECF) had the lowest value of T_g_ (80.93 °C), while the highest value of T_g_ was exhibited by PE_50_I_30_C_20_F (119.03 °C).

### 2.3. Polyesters and Copolyesters Based on FDCA and CHDM

**Figure 15:**
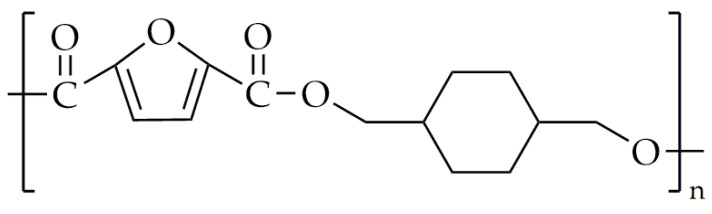
The chemical structure of PCHDMF.

There are not as many publications and patents on the synthesis of polyesters and copolyesters based on FDCA and CHDM when compared to copolyesters with isosorbide. Poly(1,4-cyclohexanedimethylene-2,5-furanoate) (PCHDMF) was synthesized by Terzopoulou et al. [83]. The chemical structure of PCHDMF is shown in Figure 15.

The PCHDMF was synthesized with the use of a modified two-step melt polycondensation. During the first step, transesterification between DMFD and CHDM occurs at 150–170 °C. After that, DMFD is added to the mixture at the molar ratio of 1/1.05, and the process is carried out in the same temperature range. The polycondensation was carried out in the temperature range of 240 °C to 260 °C under low-pressure conditions. The intrinsic viscosity of PCHDMF was 0.52 dL/g. In contrast to polyester and most copolyesters based on isosorbide, PCHDMF exhibits a crystalline structure. The peaks that were observed on WAXD diffractograms were spotted on t 2θ = 10.03, 16.7, 20.19, 22.39, and 30.81°, which is very similar to these of poly(1,4-cyclohexanedimethylene terephthalate) (PCHDMT) [88]. The T_g_ value of PCHDMF was about 74 °C, and its melting temperature (T_m_) was about 262 °C.

**Figure 16:**
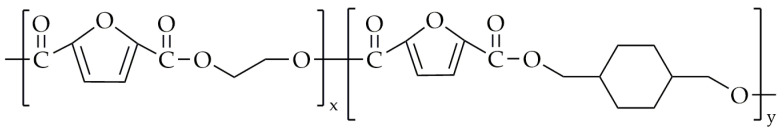
The chemical structure of PECF.

Wang et al. [89] also performed the synthesis of poly(ethylene-co-1,4-cyclohexanedimethylene-2,5-furanoate) (PECF) and PCF. The chemical structure of PECF is presented in Figure 16.

Transesterification was carried out at 180 °C until 95% of the theoretical amount of methanol was distilled. The polycondensation was performed at 240–260 °C under low-pressure conditions. The polycondensation reaction was carried out for a specific duration until the torque value of the stirrer reached the same value for all copolyesters, ensuring a consistent viscosity across all products. The CHDM was incorporated in molar fractions: 15%, 32%, 59%, and 76%. The properties of PECF can be found in Table 7**.** The incorporation of the CHDM did not result in a significantly lower intrinsic viscosity value when compared to neat PEF. Nevertheless, the intrinsic viscosity of PEF was about 0.1 dL/g higher than that of PCF, which had an intrinsic viscosity of around 0.72 dL/g. The PECF with 15 mol.% and 32 mol.% CHDM units did not exhibit a crystalline structure. At low concentrations, the CHDM units disrupt chain regularity, preventing crystallization. With a higher content of CHDM units in copolyesters and in neat PCF, the materials demonstrated a better ability to crystallize compared to PEF. However, the PEF exhibited the highest value of T_g_ (87.0 °C) when compared to copolyesters and PCF. The T_5%_ and T_d,max_ values of copolyesters did not show any significant changes when compared to the PEF, meaning that the incorporation of CHDM units does not impact the thermal stability. The E and σ_b_ values of copolyesters decreased with the higher content of CHDM units, which is probably due to the more flexible molecular chains. Thus, the ε_b_ value increased with the higher content of CHDM units (except for PE_24_C_76_F). Furthermore, the incorporation of CHDM units resulted in a greater permeability of CO_2_ and O_2_ in copolyesters when compared to the neat PEF.

**Figure 17:**
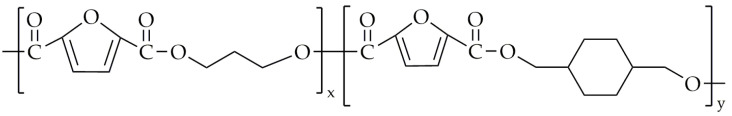
The chemical structure of PPCF.

An attempt at the synthesis of poly(propylene-co-1,4-cyclohexane-2,5-furandicarboxylate) (PPCF) was made by Jia et al. [90]. The chemical structure of PPCF is shown in Figure 17.

The transesterification was carried out at 180 °C for 3 h and at 190 °C for up to 2 h. After that, polycondensation was performed under low-pressure conditions at 250–260 °C. The polycondensation reaction was conducted until the torque value of the stirrer stabilized at the same level for all copolyesters, ensuring consistent viscosity across all products. The CHDM was incorporated in molar fractions: 20%, 40%, 57%, and 79%. The selected properties are shown in Table 8. The addition of CHDM units in some copolyesters resulted in higher intrinsic viscosity and M_n_ values. However, these values did not change significantly for most of the copolyesters. For the PPF, only a weak melting peak was detected, while the copolyesters, until a 79 mol.% of CHDM units, did not exhibit a crystalline structure. The reason for this is that CHDM units most likely enhance the crystallizability of copolyesters. The incorporation of the CHDM units also resulted in higher T_g_ values when compared to the neat PPF. Furthermore, similar to the case of PEF and PECFs, the addition of CHDM units to PPF did not affect the thermal stability of copolyesters. The incorporation of CHDM units resulted in a decrease in the E, σ_b_, and ε_b_ values. This is likely due to the higher flexibility of propylene glycol compared to CHDM.

**Figure 18:**
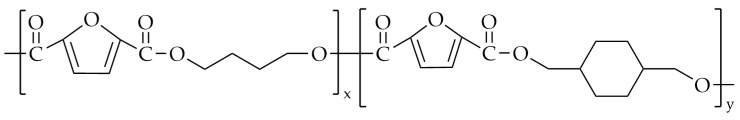
The chemical structure of PBCF.

Shen et al. [91] synthesized poly(1,4-butylene-co-1,4-cyclohexanedimethylene-2,5-furandicarboxylate) (PBCF) in 2021. The chemical structure of PBCF is presented in Figure 18.

This research group synthesized PBCF copolyesters via the traditional two-step melt polycondensation. The transesterification was conducted at 180 °C, and it lasted for about 4 h. The polycondensation was performed in the temperature range of 230 °C to 270 °C under low-pressure conditions for about 3 h. The molar content of the CHDM units in PBCF was in the range of 20 mol.% to 68 mol.%. The selected properties can be found in Table 9. The M_n_ values of copolyesters were lower when compared to the neat PBF. The incorporation of CHDM units at 20 mol.% and 40 mol.% resulted in a fully amorphous structure. Beyond 40 mol.% of CHDM units, the copolyesters exhibited a crystalline structure, with PCF demonstrating the best crystallizability. A low content of CHDM units hindered crystallization by disrupting chain regularity. In contrast, a higher content of CHDM units led to longer CHDM sequences, which enhanced regularity and promoted crystallization. The T_g_ value increased with an increasing content of CHDM units in copolyesters, even at up to 65.8 °C, which is about 27.8 °C higher than the T_g_ value of neat PBF. The thermal stability in the air and nitrogen of the copolyesters did not differ significantly from neat PBF. The higher content of CHDM units resulted in a significant increase in the E and σ_b_ values, while the ε_b_ value decreased.

Dialo et al. [92] also performed the synthesis of PBCF copolyesters. The PBCFs were also synthesized via two-step melt polycondensation. The esterification was carried out at a temperature range of 170 °C to 190 °C for about 5 h. The polycondensation was performed at 230–280 °C and lasted 5 h under low-pressure conditions. The selected properties are demonstrated in Table 10. The value of intrinsic viscosity differed significantly with the incorporation of CHDM units when compared to the neat PBF. Similarly, as in the PBFCs synthesized by Shen et al., the incorporation of CHDM units resulted in higher T_g_ values. Except for PB_80_C_20_F and PB_69_C_31_F, the T_m_ values increased alongside an increase in the CHDM units. A low content of CHDM hindered crystallization. For example, the PB_69_C_31_F copolyester was completely amorphous. With a higher content of the CHMD units, the copolyesters exhibited better values of T_d,max_ at up to 15 °C when compared to the neat PBF.

**Figure 19:**
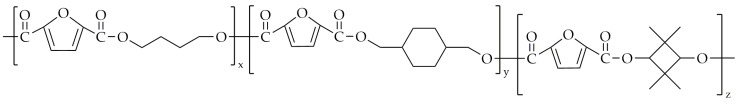
The chemical structure of PECTF.

Wang et al. [93] attempted the synthesis of poly(ethylene-co-1,4-cyclohexanedimethylene-co-2,2,4,4-tetramethyl-1,3-cyclobutanediol-2,5-furandicarboxylate) (PECTF). The chemical structure of PECTF is presented in Figure 19.

The terpolysters were synthesized via the traditional two-step melt polycondensation. The esterification was carried out in the temperature range of 180 °C to 200 °C for about 3 h. The polycondensation was performed at 245–260 °C, under low-pressure conditions, and lasted 3–4 h. The molar fraction of ethylene glycol was constant among all copolyesters (~20 mol.%). The selected properties are shown in Table 11. The incorporation of a small number of CBDO units, up to 20 mol%, resulted in higher values of M_n_. However, a further decrease in CHDM units and an increase in CBDO units resulted in significantly lower M_n_ values. The M_n_ value of PECTF-0 was two times higher than the M_n_ value of PECTF-53. The T_g_ values of copolyesters increased with the increase in CBDO units. PECTF-53 had the highest T_g_ value, at about 105.7 °C, which was 25.2 °C higher when compared to PECT-0 (80.3 °C). However, only copolyesters with small amount of CBDO units (up to 15 mol.%) had a crystalline structure. The increase in T_g_ values and the reduction in or complete loss of crystalline structures were attributed to the incorporation of CBDO units. This is due to the increased rigidity and stiffness of the molecular chains resulting from the incorporation of CBDO units, which hindered the crystallization process and increased the T_g_ values of the copolyesters. Nevertheless, the increase in CBDO units did not affect the thermal stability of the copolyesters. With an increase in the CBDO units and a decrease in the CHDM units, the E and σ_b_ values generally increased, except for PECTF-53, which exhibited the lowest E and σ_b_ values. This is likely due to the very low M_n_ value of this particular copolyester. The highest value of σ_b_ was exhibited by PECTF-45, about 88 MPa, which was 13 MPa higher compared to PECTF-0. The ε_b_ value decreased with an increase in the CBDO units. This was especially visible for PECTF-53, which had ε_b_ value of around 5%, which was 188% lower than PECTF-0. This is likely due to the low M_n_ value of PECTF-53 and the increased chain stiffness resulting from the addition of CBDO units.

### 2.4. Polyesters and Copolyesters Based on FDCA and CBDO

**Figure 20:**
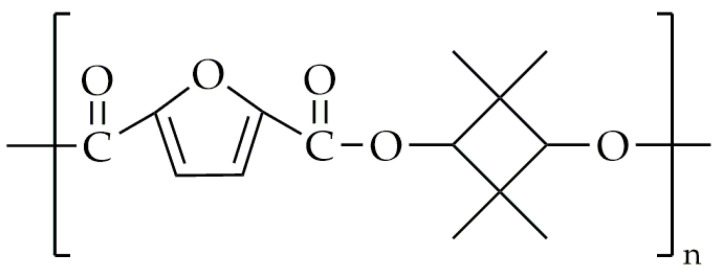
The chemical structure of PTF.

The only source available regarding the synthesis of poly(2,2,4,4-tetramethyl-1,3-cyclobutanediol-2,5-furandicarboxylate) (PTF) is the European patent EP3235848A1 by Van ES et al. [94]. The chemical structure of PTF is presented in Figure 20.

The PTF was synthesized via the traditional two-step melt polycondensation. The transesterification was performed for 12 h at 160 °C. The second step was carried out at 215–220 °C and lasted 3 h under low-pressure conditions. The synthesized polyester exhibited a T_g_ value of 124 °C, which was higher when compared to the T_g_ value of PEF or PCF but lower than that of PIF. Additionally, the M_n_ value of the polymer was approximately 10,000 g/mol. Unfortunately, no further data regarding the polymer’s structural or compositional characteristics were provided in the patent.

**Figure 21:**
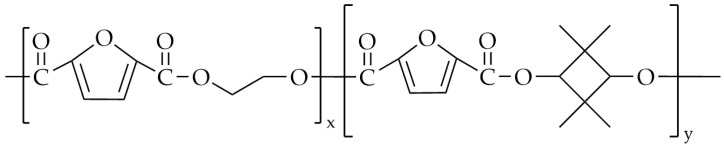
The chemical structure of PETF.

Wang et al. [95] attempted the synthesis of poly(ethylene-co-2,2,4,4-tetramethyl-1,3-cyclobutanediol 2,5-furandicarboxylate) (PETF). The two-step melt polycondensation was carried out in a 500 mL three-necked round-bottom flask. The esterification was conducted at 175–180 °C and lasted 4 h. The polycondensation was performed at 235–240 °C under low-pressure conditions. The second step lasted until the torque value reached the same fixed value for all materials. The scheme of the synthesis is presented in Figure 21.

Selected properties of the materials are presented in Table 12. The incorporation of the CBDO units resulted in a decrease in the value of intrinsic viscosity, even at up to 0.21 dL/g, when compared to the neat PEF. The M_n_ value did not change significantly with a higher CBDO content among the copolyesters, exhibiting a value of around 18,000 g/mol. The PETFs had a higher value of T_g_ when compared to PEF, but their structure was completely amorphous. The reason for this is the higher rigidity of CBDO when compared to ethylene glycol. The thermal stability of PEF and PETFs was comparable, with T_d,5%_ differing by approximately 9 °C and T_d,max_ varying by only about 5 °C. The increase in the content of CBDO units resulted in an increase in the E and σ_b_ values, while the ε_b_ value remained unchanged. This was attributed to the increased rigidity of the molecular chain, which had resulted from the introduction of CBDO units.

**Figure 22:**
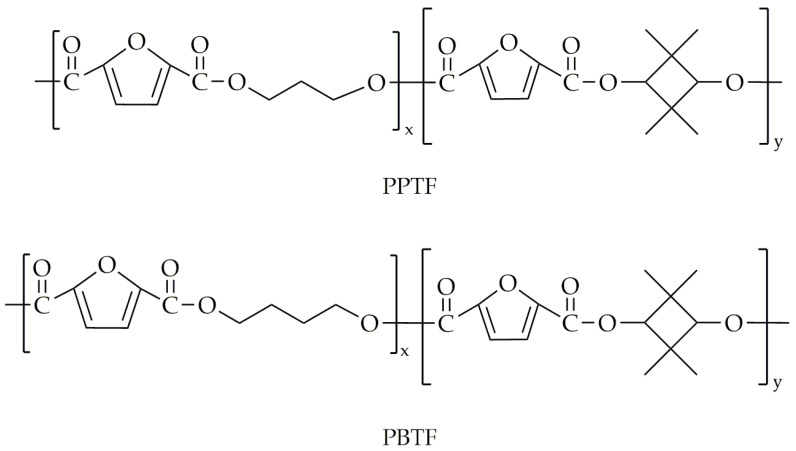
The chemical structure of PPTF and PBTF.

This same research group undertook a subsequent synthesis of various polyesters [96], including PEF and PETFs, along with PPF and its copolyesters, poly(propylene-co-2,2,4,4-tetramethyl-1,3-cyclobutanediol 2,5-furandicarboxylate)s (PPTF)s. Additionally, they synthesized PBF and its copolymers, poly(tetramethylene-co-2,2,4,4-tetramethyl-1,3-cyclobutanediol 2,5-furandicarboxylate)s (PBTF)s. The chemical structures of PPTF and PBTF are presented in Figure 22. Similar to the previous attempt, the two-step melt polycondensation was carried out in a 500 mL three-necked round-bottom flask. The transesterification was performed at 180 °C and lasted 4 h. During the second step, polycondensation was conducted at a temperature range of 230 to 245 °C under low-pressure conditions. The polycondensation reaction was conducted until the torque value of the stirrer stabilized at the same level for all copolyesters, ensuring consistent viscosity across all products. The CBDO units were incorporated in 10% and 18% molar fractions. The selected properties are presented in Table 13.

In the case of PETFs and PPTFs, one can observe a decrease in the intrinsic viscosity and M_n_ value when compared to PEF and PPF, respectively, with the exception of PP_90_T_10_F. However, the incorporation of CBDO units had no notable effect on the intrinsic viscosity and M_n_ values of PBTF compared to PBF. The T_g_ value of copolyesters increased with a higher content of CBDO units. The highest T_g_ value was exhibited by PE_82_T_18_F (91.1 °C), while the lowest was exhibited by PBF (39.0 °C). Moreover, the incorporation of CBDO units caused a completely amorphous structure in copolyesters. The higher T_g_ value and lack of crystalline structure were attributed to the rigid structure of CBDO units, which hindered the movement of molecular chains. The thermal stability alongside the materials did not significantly differ with the addition of CBDO units. The incorporation of CBDO units resulted in higher values of E and σ_b_ of the copolyesters when compared to the neat polyesters that they were based on. The difference in σ_b_ value between the copolyester and neat polyester was even up to 18 MPa. Nevertheless, the ε_b_ value decreased with a higher content of CBDO units in copolyesters. This alteration was not notable for PEF and its copolyesters. The most significant impact of incorporating CBDO units into the copolyesters was observed for PBF and its copolyesters. Specifically, PB_82_T_18_F exhibited an ε_b_ that was around 80% lower than that of neat PBF. The E and σ_b_ values, along with the lower ε_b_ value of the copolyesters, compared to their respective neat polyesters, could be attributed to the increased rigidity of the molecular chains resulting from the incorporation of CBDO units.

## 3. Conclusions and Outlook

**Figure 23:**
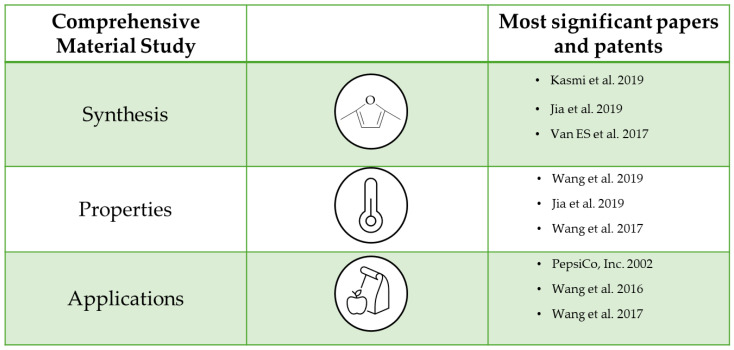
The most important papers and patents about comprehensive copolymer studies [81,82,85,89,90,94,95].

The commercialization of polyesters synthesized from bio-based raw materials is crucial for environmental safety. Thus, a further modification of bio-based polyesters to tailor their properties is a natural progression, thereby expanding their applicability in the various market segments that require specific material characteristics. The most important papers are organized and presented in Figure 23.

A summary of the comparison between copolyesters and FDCA-based counterparts can be found in Table 14. Modifying bio-based polyesters with rigid diols, like isosorbide, CHDM, or CBDO, has been proven to be effective. The incorporation of rigid diols resulted in an increase in the T_g_ value, except for PECF copolymers. Moreover, the addition of rigid diols affected the mechanical properties of copolymers, with most of the copolyesters exhibiting higher values of E and σ_b_ with an increase in the rigid diol units. This is due to the increased stiffness of the molecular chains in copolyesters, resulting from the incorporation of rigid diols. This modification leads to superior thermomechanical properties, making these polyesters suitable for applications in sectors such as packaging and the automotive industry. Despite the advantages of the incorporation of rigid diols, the disadvantage of isosorbide and CBDO is their low reactivity, which results in the low M_n_ values of copolyesters. However, the incorporation of CHDM did not significantly affect the M_n_ values. In addition, only the copolyesters with CHDM units exhibited an increase in X_c_ values when compared to the other copolyesters based on the rigid diols. Nevertheless, the copolyesters with CHDM exhibited lower E values when compared to the neat polyesters. The incorporation of various rigid diols has caused alterations in a range of properties with different magnitudes. Therefore, the use of different rigid glycols must be considered in order to obtain customized properties for special applications. The continuous research and development in FDCA-based polyesters and copolyesters will lead to these materials becoming more affordable and widely available in the future. Copolyesters based on FDCA and isosorbide could be used for hot fill packaging due to their high value of T_g_. Additionally, the incorporation of the CBDO into FDCA-based polyesters enhances their mechanical properties and makes them suitable for application in the automotive industry. The superior O_2_ barrier properties of the copolyesters based on CHDM and FDCA, when compared to the FDCA-based counterparts, make them suitable for the production of films and packaging. However, furan can be metabolically activated upon absorption by the gastrointestinal tract [97]. Further studies need to be carried out for the assessment of furan toxicity on human health.

## Figures and Tables

**Table 1:** Selected properties of PEIF synthesized in a glass and stainless-steel reactor.

In a 200 mL Glass Reactor				
IS_NMR_ [mol%]	M_n_ [g/mol]	M_w_ [g/mol]	PDI	T_g_ [°C]
14.6	14,900	26,750	1.8	80
16.4	4600	17,100	3.7	87
25	5050	15,250	3.0	83
**In a 2 L Stainless-Steel Reactor**				
13.2	37,300	60,050	1.6	99
14.4	27,300	50,950	1.9	94
18.6	30,050	66,650	2.0	102

IS_NMR_—calculated content of isosorbide, M_n_—number-average molecular weight, M_w_—weight-average molecular weight, PDI—polydispersity index, T_g—_glass transition value.

**Table 2:** Selected properties of PEIF synthesized by the method in WO2015142181A1.

Isosorbide Is Utilized in the Initial Step of the Process				
EG/IS	IS_NMR_ [mol%]	M_n_ [g/mol]	PDI	T_g_ [°C]
1.2	55.0	6570	1.99	126
1.6	43.2	5900	2.33	115
2.0	41.3	7100	2.40	111
**Ethylene Glycol Is Utilized in the Initial Step of the Process**				
1.0	22.3	8430	2.23	101
1.7	14.7	11,260	2.29	98
3.0	8.8	9180	2.33	90

EG/IS—ratios of ethylene glycol/isosorbide in the initial mixture, IS_NMR_—calculated content of isosorbide, M_n_—number-average molecular weight, PDI—polydispersity index, T_g_—glass transition value.

**Table 3:** Selected properties of PBIF synthesized in a glass and stainless-steel reactor.

Material	IS_NMR_ [mol%]	M_n_ [g/mol]	PDI	T_g_ [°C]	E [MPa]	ε_b_ [%]
PBF	-	-	-	42.3	1502 ± 101	685 ± 32
PB_80_I_20_F	15.9	19,100	1.96	55.3	1464 ± 100	435 ± 31
PB_70_I_30_F	24.7	17,700	2.01	68.9	1488 ± 91	306 ± 28
PB_60_I_40_F	37.5	15,900	2.04	87.2	1408 ± 54	173 ± 12
PB_50_I_50_F	47.6	13,100	1.94	103.1	1471 ± 61	46 ± 7
PB_40_I_60_F	55.3	9300	1.98	110.4	1590 ± 42	32 ± 2
PB_30_I_70_F	65.2	-	-	130.0	1735 ± 68	28 ± 2
PB_20_I_80_F	76.3	-	-	150.6	1900 ± 60	15 ± 3

IS_NMR_—calculated content of isosorbide, M_n_—number-average molecular weight, PDI—polydispersity index, T_g_—glass transition value, E—Young’s modulus, ε_b_—elongation at break.

**Table 4:** Properties of selected PHF and PHIF.

Material	IS_NMR_ [mol%]	[η] [dL/g]	T_g_ [°C]	T_d,5%_ [°C]
PHF	-	0.40	7	339.7
PH_97_I_3_F	2.4	0.41	10	365.5
PH_90_I_10_F	12.8	0.58	20	368.5
PH_70_I_30_F	27.7	0.37	34	370.3
PH_50_I_50_F	47.9	0.27	58	369.3
PH_30_I_70_F	67	0.32	101	354.4
PH_10_I_90_F	88.3	0.23	135	373.5

IS_NMR_—calculated content of isosorbide, [η]—intrinsic viscosity, T_g_—glass transition value, T_d,5%—_decomposition temperatures at a 5% weight loss.

**Table 5:** Selected properties of PDF and PDIF.

Material	IS_NMR_ [mol%]	M_n_ [g/mol]	T_g_ [°C]	X_c_ [%]	T_d,5%_ [°C]	E [MPa]
PDF	-	-	1	49.3	406.1	201.9 ± 15
PD_95_I_5_F	7	21,600	−1.2	29.2	408.7	284.5 ± 7.5
PD_90_I_10_F	12	21,500	−0.1	28.4	411.7	268.0 ± 5
PD_85_I_15_F	15.7	25,400	3.7	25.9	412.8	558.6 ± 8
PD_80_I_20_F	19	15,900	6.3	26.7	406.3	165.1 ± 31
PD_70_I_30_F	28	11,500	9.7	25.2	404.9	13.8 ± 3.0
PD_60_I_40_F	39	17,200	20.6	23.2	408.6	88.5 ± 36

IS_NMR_—calculated content of isosorbide, M_n_—number-average molecular weight, T_g_—glass transition value, X_c_—degree of crystallinity, T_d,5%_—decomposition temperatures at a 5% weight loss, E—Young’s modulus.

**Table 6:** Selected properties of PECF and PEICF.

Material	IS/CHDM_NMR_ [mol%]	M_n_ [g/mol]	T_g_ [°C]
PE_50_C_50_F	-/51	19,300	80.93
PE_50_I_10_C_40_F	12/42	18,700	90.34
PE_50_I_20_C_30_F	23/33	19,600	102.43
PE_50_I_20_C_30_F	49/19	16,400	119.03

IS/CHDM_NMR_—calculated content of isosorbide and CHDM, M_n_—number-average molecular weight, T_g_—glass transition value.

**Table 7:** Selected properties of PEF, PCF, and PECFs.

Material	CHDM_NMR_ [mol%]	[η] [dL/g]	T_g_ [°C]	T_m_ [°C]	T_d,5%_ [°C]	E [MPa]
PEF	-	0.82	87.0	211.9	372.3	2800 ± 15
PE_85_C_15_F	15.1	0.77	84.9	-	368.7	2300 ± 7.5
PE_68_C_32_F	32.0	0.86	83.8	-	367.3	2200 ± 80
PE_41_C_59_F	59.2	0.92	81.7	206.5	368.0	1740 ± 5
PE_24_C_76_F	75.9	0.79	80.6	225.4	365.0	1760 ± 8
PCF	100.0	0.72	79.5	262.7	371.3	2100 ± 31

CHDM_NMR_—calculated content of CHDM, [η]—intrinsic viscosity, T_g_—glass transition value, T_m_—value of melting temperature, T_d,5%—_decomposition temperatures at a 5% weight loss, E—Young’s modulus.

**Table 8:** Selected properties of PPF, PCF, and PECFs.

Material	CHDM_NMR_ [mol%]	[η] [dL/g]	T_g_ [°C]	T_m_ [°C]	T_d,5% in N2_ [°C]	E [MPa]
PPF	-	0.74	56.5	173.4	376	2460 ± 280
PP_80_C_20_F	20	0.78	59.4	-	378	2100 ± 90
PE_60_C_40_F	40	0.68	64.2	-	380	1992 ± 70
PE_43_C_57_F	57	0.81	68.7	193.5	379	1925 ± 60
PE_21_C_79_F	79	0.71	73.7	231.8	382	2106 ± 80
PCF	100.0	0.62	79.8	268.5	381	2100 ± 200

CHDM_NMR_—calculated content of CHDM, [η]—intrinsic viscosity, T_g—_glass transition value, T_m_—value of melting temperature, T_d,5%_—decomposition temperatures at a 5% weight loss, E—Young’s modulus.

**Table 9:** Selected properties of PBF, PCF, and PBCFs.

Material	CHDM_NMR_ [mol%]	M_n_ [g/mol]	T_g_ [°C]	T_d,max% in N2_ [°C]
PBF	-	48,900	38.0	403
PB_80_C_20_F	20	40,800	47.7	397
PB_60_C_40_F	40	38,700	53.7	397
PB_47_C_53_F	53	44,200	60.9	397
PB_32_C_68_F	68	38,400	65.8	396
PCF	100	25,900	87.9	413

CHDM_NMR_—calculated content of CHDM, M_n_—number-average molecular weight, T_g_—glass transition value, T_d,5%_—decomposition temperatures at a 5% weight loss.

**Table 10:** Selected properties of PBF, PCF, and PBCFs.

Material	CHDM_NMR_ [mol%]	[η] [dL/g]	T_g_ [°C]	T_d,max% in N2_ [°C]
PBF	-	0.77	39.7	373.0
PB_90_C_10_F	20	0.49	45.7	380.6
PB_80_C_20_F	31	0.93	54.4	381.6
PB_70_C_30_F	52	0.40	61.3	382.1
PB_60_C_40_F	61	0.63	68.0	387.4
PB_50_C_50_F	70	0.25	74.4	388.0
PCF	100	-	87.4	390.7

CHDM_NMR_—calculated content of CHDM, [η]—intrinsic viscosity, T_g_—glass transition value, T_d,5%_—decomposition temperatures at a 5% weight loss.

**Table 11:** Selected properties of PECTF copolyesters.

Material	CHDM/CBDO [mol%]	CHDM/CBDO_NMR_ [mol%]	M_n_ [g/mol]	T_g_ [°C]	T_d,max% in N2_ [°C]	E [MPa]	ε_b_ [%]
PECTF-0	85/0	81/0	35,100	80.3	414	1820 ± 50	193 ± 8
PECTF-8	75/10	73/8	36,000	83.5	417	1910 ± 60	186 ± 11
PECTF-15	65/20	66/15	36,800	87.9	420	1950 ± 60	168 ± 7
PECTF-25	55/30	55/25	24,100	90.9	417	2020 ± 30	126 ± 9
PECTF-35	45/40	45/35	24,700	96.0	417	2060 ± 30	95 ± 8
PECTF-45	35/50	35/45	22,200	103.1	416	2140 ± 60	67 ± 3
PECTF-53	25/60	25/53	17,300	105.7	416	1780 ± 90	5 ± 1

CHDM/CBDO—ratios of CHDDM/CBDO in the initial mixture, CHDDM/CBDO_NMR_—calculated content of CHDM and CBDO, M_n_—number-average molecular weight, T_g_—glass transition value, T_d,5%_—decomposition temperatures at a 5% weight loss, E—Young’s modulus, ε_b_—elongation at break.

**Table 12:** Selected properties of PEF and PETFs.

Material	CBDO_NMR_ [mol%]	[η] [dL/g]	T_g_ [°C]	T_d,5% in N2_ [°C]	E [MPa]	ε_b_ [%]
PEF	-	0.92	87.0	372.3	2800 ± 120	5 ±1
PE_96_T_4_F	5	0.71	88.9	372.0	3000 ± 130	6 ± 1
PE_90_T_10_F	14	0.79	90.9	368.7	3100 ± 100	6 ± 5
PE_85_T_15_F	21	0.72	92.1	364.7	3400 ± 160	5 ± 0.1
PE_77_T_23_F	30	0.71	94.3	363.6	3500 ± 100	4 ± 0.2

CBDO_NMR_—calculated content CBDO, [η]—intrinsic viscosity, T_g_—glass transition value, T_d,5%_—decomposition temperatures at a 5% weight loss, E—Young’s modulus, ε_b_—elongation at break.

**Table 13:** Selected properties of PEF, PPF, PBF, and copolyesters based on them.

Material	CBDO_NMR_ [mol%]	[η] [dL/g]	T_g_ [°C]	T_d,5% in N2_ [°C]	E [MPa]	ε_b_ [%]
PEF	-	0.92	87.0	365	2800 ± 120	5 ±1
PE_90_T_10_F	10.3	0.79	90.9	368	3100 ± 100	9 ± 5
PE_82_T_18_F	18.2	0.74	91.1	369	3300 ± 100	4 ± 1
PPF	-	0.88	55.5	367	2700 ± 30	50 ± 7
PP_90_T_10_F	9.8	0.93	61.1	370	2750 ± 20	56 ± 11
PP_82_T_18_F	17.8	0.76	63.5	361	2800 ± 40	30 ± 10
PBF	-	0.98	39.0	367	2000 ± 30	290 ± 6
PB_90_T_10_F	9.6	0.96	42.5	368	2100 ± 80	274 ± 10
PB_82_T_18_F	17.9	0.92	43.5	365	2200 ± 60	220 ± 18

CBDO_NMR_—calculated content of CBDO, [η]—intrinsic viscosity, T_g_—glass transition value, T_d,5%_—decomposition temperatures at a 5% weight loss, E—Young’s modulus, ε_b_—elongation at break.

**Table 14:** A comparison of copolyesters to FDCA-based polyester counterparts.

Materials	Advantages	Disadvantages	Industrial Application
Polyesters and copolyesters based on FDCA and isosorbide.	Higher value of T_g_ and higher value of E.	Lower value of Mn and lower value of ε_b_.	Hot fill packaging.
Polyesters and copolyesters based on FDCA and CHDM.	Generally higher value of X_c_, better O_2_ barrier properties, high transparency,	Lower value of E and worst CO_2_ barrier properties.	Films and containers.
Polyesters and copolyesters based on FDCA and CBDO.	higher value of E, higher value of σ_b_, and mildly lower values of [η].	Worst CO_2_ and O_2_ barrier properties.	Automotive and packaging.
